# Dissecting Age-Stratified Immunity to Different Dengue Virus Serotypes and Zika Viruses Among Children in a Highly Endemic Region in Sri Lanka

**DOI:** 10.1093/ofid/ofag224

**Published:** 2026-04-18

**Authors:** Shyrar Tanussiya Ramu, Madushika Dissanayake, Chamini Kanatiwela de Silva, Naduni Dasanthi, Amaya Gunaratne, Saubhagya Danasekara, Laksiri Gomes, Chandima Jeewandara, Nicole L Achee, John P Grieco, H Asita de Silva, D S Anoja F Dheerasinghe, Thomas W Scott, Amy C Morrison, Hasitha Aravinda Tissera, Gathsaurie Neelika Malavige

**Affiliations:** Allergy Immunology and Cell Biology Unit, Department of Immunology and Molecular Medicine, University of Sri Jayewardenepura, Nugegoda, Sri Lanka; Allergy Immunology and Cell Biology Unit, Department of Immunology and Molecular Medicine, University of Sri Jayewardenepura, Nugegoda, Sri Lanka; RemediumOne, Colombo, Sri Lanka; Allergy Immunology and Cell Biology Unit, Department of Immunology and Molecular Medicine, University of Sri Jayewardenepura, Nugegoda, Sri Lanka; Allergy Immunology and Cell Biology Unit, Department of Immunology and Molecular Medicine, University of Sri Jayewardenepura, Nugegoda, Sri Lanka; Allergy Immunology and Cell Biology Unit, Department of Immunology and Molecular Medicine, University of Sri Jayewardenepura, Nugegoda, Sri Lanka; Allergy Immunology and Cell Biology Unit, Department of Immunology and Molecular Medicine, University of Sri Jayewardenepura, Nugegoda, Sri Lanka; Allergy Immunology and Cell Biology Unit, Department of Immunology and Molecular Medicine, University of Sri Jayewardenepura, Nugegoda, Sri Lanka; Department of Biological Sciences, Eck Institute for Global Health, University of Notre Dame,Notre Dame, Indiana, USA; Department of Biological Sciences, Eck Institute for Global Health, University of Notre Dame,Notre Dame, Indiana, USA; Clinical Trials Unit, Faculty of Medicine, University of Kelaniya, Kelaniya, Sri Lanka; National Dengue Control Unit, Ministry of Health, Colombo, Sri Lanka; Department of Entomology and Nematology, University of California, Davis, Davis, California, USA; Department of Pathology, Microbiology and Immunology, School of Veterinary Medicine, University of California, Davis, Davis, California, USA; Epidemiology Unit, Ministry of Health, Colombo, Sri Lanka; Allergy Immunology and Cell Biology Unit, Department of Immunology and Molecular Medicine, University of Sri Jayewardenepura, Nugegoda, Sri Lanka

**Keywords:** Dengue, Luminex, seroprevalence, serotypes, Zika

## Abstract

**Background:**

Determining serotype-specific, age-stratified dengue virus (DENV) and Zika virus (ZIKV) seroprevalence rates is crucial for implementing vaccines and vector control strategies. Therefore, we sought to assess the age-stratified seroprevalence of monotypic or multitypic exposure in a community cohort in Sri Lanka.

**Methods:**

DENV-specific serostatus was assessed in 4161 children aged 4 to 16 years, using an in-house DENV-specific IgG enzyme-linked immunosorbent assay (ELISA), and results were compared with those of a widely used commercial assay (Panbio Dengue IgG Indirect ELISA). We also used a multiplexed, microsphere-based serological assay to characterize monotypic vs multitypic responses and to differentiate exposure rates to different DENV serotypes and ZIKV in a subcohort of children (n = 604).

**Results:**

By IgG ELISA, DENV seropositivity was 72.34% (3010/4161), and the seropositivity rate significantly increased with age (Spearman *r* = 1.0, *P* = .003). The estimated force of infection was 0.16 (95% credible interval, 0.14–0.17). Of the 604 individuals tested by Luminex, 258 (42.7%) had a monotypic dengue response, whereas 209 (34.9%) had a multitypic response. Moreover, 100 (16.5%) had evidence of a past infection to Zika, while 20 (3.33%) children had antibodies only to ZIKV. Of the 258 individuals with evidence of a monotypic response to DENV, DENV2 (56.83%) and DENV1 (30.57%) accounted for the most infections. There was an inverse correlation between exposure to ZIKV and age (Spearman *r* = −0.72, *P* = .007).

**Discussion:**

An overall 72.3% of children were seropositive for dengue, with 42.7% having been infected with only 1 DENV in the past. The data suggest that prior immunity to DENV may reduce the risk of ZIKV infection, which should be further assessed.

Dengue is the most rapidly emerging mosquito-borne viral infection, with 13 million cases in 2024 [1]. The burden attributed to dengue is predicted to rise due to climate change, rapid urbanization, and population displacements [2] and has been named in the World Health Organization’s R&D Blueprint as a pathogen of pandemic potential [3]. Although it has caused outbreaks in many tropical and subtropical countries for >5 decades, few innovations have been developed in vector control strategies, diagnostics, treatments, and development of vaccines. Although 2 vaccines have been licensed for prevention of dengue, CYD-TDV had limited efficacy against some dengue virus (DENV) serotypes, with a potential to cause an increase in hospitalization and severe dengue in individuals who were dengue naive [4]. The most recently licensed vaccine, TAK-003 (Qdenga), is again not equally effective against all DENV serotypes [4]. Furthermore, the World Health Organization has recently recommended that TAK-003 be used in children aged 6 to 16 years in high-transmission settings, while not recommending it for use in low- or moderate-transmission settings [5]. Therefore, to implement vaccine strategies and integrated vaccination–vector control strategies [6], it is crucial to understand the dynamics of dengue transmission in different settings in endemic countries.

Sri Lanka has been experiencing regular dengue outbreaks since 1989, with a gradual rise in the number of cases in recent years [7]. Although dengue outbreaks are reported from all over the country, 50% of the cases are from the Western province, in the Colombo and Gampaha districts [7]. Based on population studies conducted in 2003, 2013, 2017, 2022, and 2024 in the Colombo district [7–10], age seroprevalence curves have been gradually shifting to higher seroprevalence rates in younger groups, consistent with a rise in dengue transmission [7, 8]. However, our recent island-wide, age-stratified dengue seroprevalence study demonstrated significant heterogeneity across districts in Sri Lanka, with differences among urban, rural, and estate areas [11]. For instance, while the estimated force of infection (FOI) for the city of Colombo is 0.149, the FOI for suburban areas in Colombo was an estimated 0.068 [10]. For some districts in Sri Lanka, the FOI has been estimated to be low: 0.011 in Badulla, which is predominantly an estate region, and 0.012, 0.014, and 0.013 in Kurunegala, Matara, and Kandy, respectively, which are predominantly rural areas [10].

Although age-stratified seroprevalence studies give some indication of the burden of infection in a community, the assays used in these studies (ie, DENV-specific IgG detection assays) detect cross-reactive antibodies for other flaviviruses and cannot differentiate between a past infection due to 1 DENV serotype (monotypic infections) and infection due to several DENV serotypes (multitypic infections) [8, 12, 13]. Differentiating monotypic vs multitypic exposure to DENV would provide better granularity in understanding the transmission dynamics of dengue infections. Furthermore, differentiating cross-reactive flavivirus antibody responses is important to understand how population immunity to different flaviviruses affects the emergence and spread of dengue outbreaks. For instance, it has been shown that infection with the Zika virus (ZIKV) enhances dengue disease severity during subsequent dengue infections [14]. As IgG antibodies to DENV and ZIKV highly cross-react with each other [15], conventional assays that measure either DENV- or ZIKV-specific IgG would not provide reliable information regarding past infection with DENV serotypes or ZIKV. Although surveillance for the presence of ZIKV has not been routinely carried out in Sri Lanka, a recent study showed that ZIKV was identified in 1% of individuals presenting with dengue-like illness from 2017 to 2019 [16]. Given that Zika outbreaks have been reported in many countries, including South Asia, it would be important to determine exposure rates to ZIKV in age groups in Sri Lanka [17, 18] to understand how infection with different flaviviruses may modulate transmission of dengue and clinical disease outcomes.

To understand the dengue transmission dynamics, the rates of monotypic or multitypic DENV infection, as well as past infection with ZIKV in Sri Lanka, we studied the age-stratified seroprevalence in a large cohort of children and proceeded to use a multiplexed, microsphere-based serologic assay (Luminex assay) to differentiate exposure rates to DENV serotypes and ZIKV in an endemic region in Sri Lanka.

## MATERIALS AND METHODS

### Study Participants

This study was carried out under the AEGIS study (Advancing Evidence for the Global Implementation of Spatial Repellents), which aims to generate evidence for implementation of novel vector control strategies.

Children (N = 4161) aged 4 to 16 years were recruited from 3 divisions of the Medical Officer of Health (ie, MOH areas) from the Gampaha district, Sri Lanka, which reported a high incidence of dengue. An MOH area refers to a specific administrative division that is overseen by an MOH, who is responsible for implementing and managing public health services. This study was conducted as part of baseline studies for a vector intervention trial with a randomized controlled design with 30 clusters (10 from each MOH area).

### Sampling Technique

All houses within the selected clusters that gave their consent were mapped, and houses with children aged 4 to 16 years were offered enrollment. Enrollment continued in each cluster until the target sample size of 130 per cluster was achieved. Blood samples were collected from these children by trained health care professionals using standard venipuncture procedures within a period of 5 months from June 2023 to October 2023 [6]. The collection was carried out in a controlled and sterile environment to ensure the safety and comfort of the children.

### Ethical Approval

Ethical approval for the study was obtained from the Ethics Review Committee, University of Kelaniya, Sri Lanka, and approved by the institutional review boards of the World Health Organization’s Ethical Review Committee (ERC.0003619) and the University of Norte Dame (21-05-6629). All participants were recruited into the study after the provision of informed consent from their parents or guardians, with assent obtained from children where appropriate.

### Development and Optimization of an In-house Dengue IgG Enzyme-Linked Immunosorbent Assay to Determine Serostatus

An in-house enzyme-linked immunosorbent assay (ELISA) was developed and optimized to measure the presence of DENV-specific IgG antibodies in the children. The in-house ELISA was validated by foci reduction neutralization assays [19] as the gold standard and compared with a commercial assay, the Panbio Dengue IgG Indirect ELISA (henceforth, Panbio assay), which has been widely used to detect the serostatus of individuals in community serosurveillance studies in Sri Lanka and elsewhere. Details of the development and validation of the in-house DENV-specific IgG assay are given in the Supplementary Methods.

### Multiplexed, Microsphere-Based Serologic Assay to Determine Infection History

A multiplexed, microsphere-based serological assay (Luminex assay) was carried out as previously described [20] to determine if children who were DENV seropositive had been exposed to only 1 infection (monotypic), many infections (multitypic), or Zika. Developed by Prof Lakshmanane Premkumar (Department of Microbiology and Immunology, School of Medicine, University of North Carolina), this assay uses antigens of the most variable region of DENV and ZIKV (EDIII) to differentiate antibody responses to DENV serotypes and ZIKV [20]. Due to limitations in resources, these assays were carried out on 20% of the samples (n = 604) that gave a positive response with the in-house DENV-specific IgG assay. We randomly selected children using stratification by age group and gender to ensure representation across these categories. To ensure equal representativeness of all age groups and both genders, we used a stratified random sampling procedure. Stratification was done across districts (Kelaniya, Negombo, Wattala), age categories (4–7, 8–11, and 12–16 years), and gender. Within each stratum, 20% of the available positive samples were randomly selected. This ensured that the subcohort retained the same proportional distribution of age and gender as the full seropositive population.

**Figure 1.**
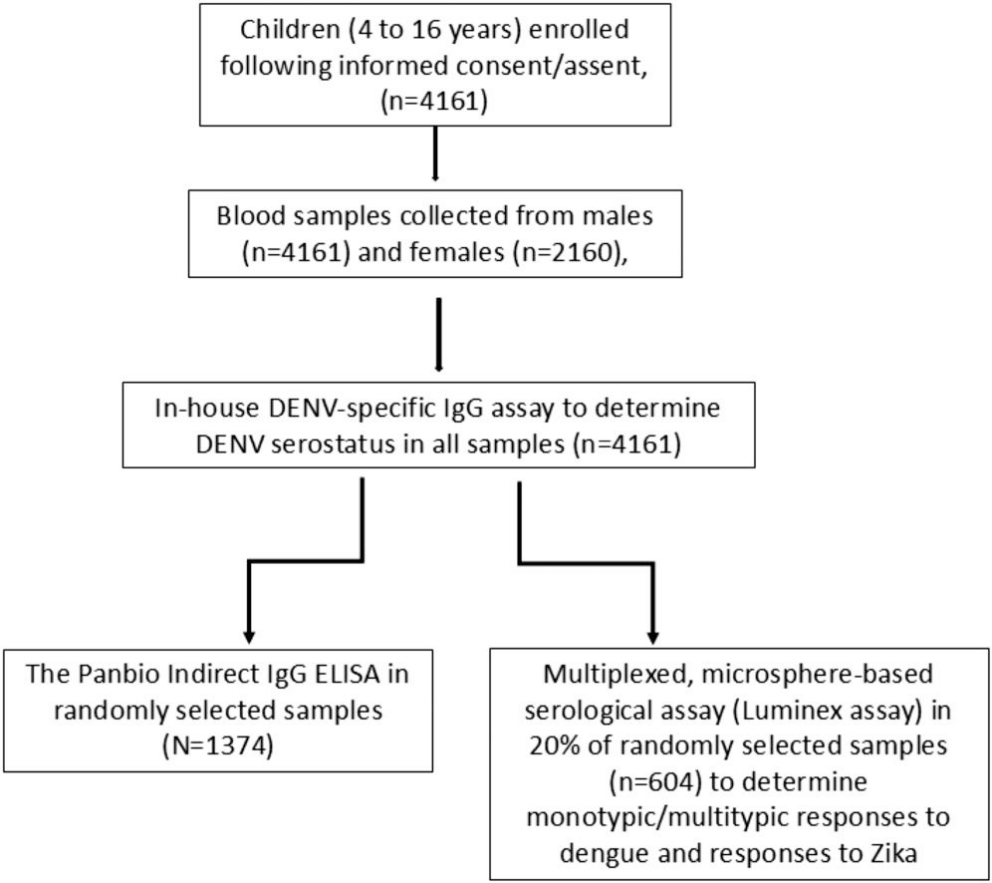
Study summary flowchart. A summary of participant selection, sample processing, and assay allocation used in this study. Samples are processed and then subjected to assays. Abbreviations: DENV, dengue virus; ELISA, enzyme-linked immunosorbent assay.

See details of the Luminex methodology and statistical analysis in the Supplementary Methods. Participant selection, sample processing, and assay allocation are summarized in Figure 1.

## RESULTS

### Age-Stratified Dengue Seroprevalence of the Children

**Figure 2.**
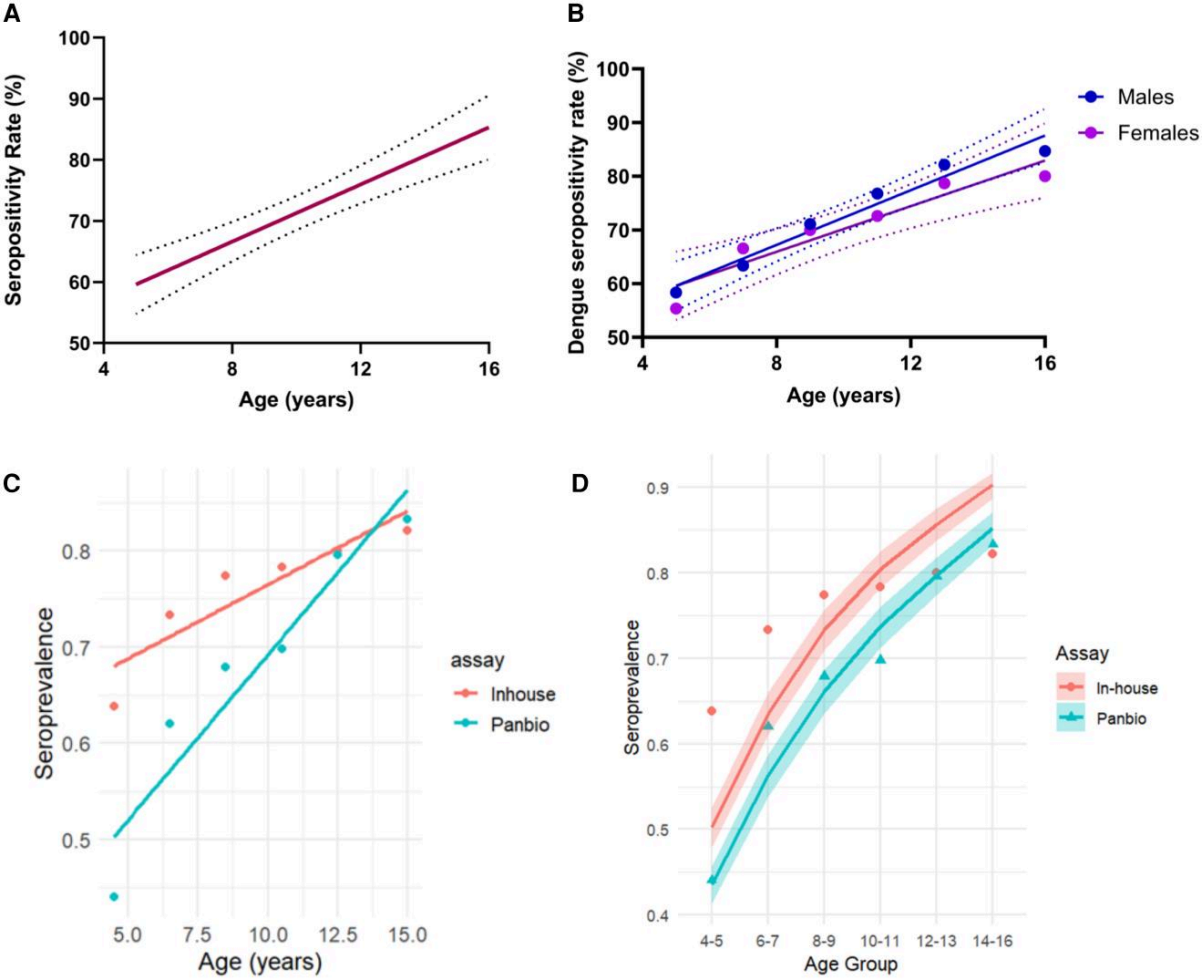
Age-stratified seropositivity rates in children. *A*, Dengue IgG levels were measured in 2160 males and 2001 females between the ages of 4 and 16 years (n = 4161) from the Gampaha district by an in-house DENV-specific IgG ELISA, and dengue seropositivity rates were correlated with the age. *B*, Dengue antibody positivity rates were also correlated with age in males (blue) and females (purple). Dotted lines indicate the 95% confidence intervals. The dengue IgG seropositivity rates were also determined for the in-house ELISA and the commercial Panbio Dengue IgG Indirect ELISA (n = 1374). Solid lines represent the best-fit simple linear regression models for each assay, showing the relationship between age and seropositivity. *C*, A statistically significant interaction between age and assay type was observed (*P* = .01), indicating differences in the age-seroprevalence slopes between assays. *D*, The observed and estimated age-stratified seroprevalence per the in-house DENV-specific IgG ELISA and the commercial Panbio assay was carried out on 1374 children. Dots and triangles represent observed seropositivity proportions by age group, while shaded curves represent bayesian model–based estimates of seroprevalence with 95% credible intervals. The force of infection was estimated via the bayesian catalytic model. DENV, dengue virus; ELISA, enzyme-linked immunosorbent assay.

Out of 4161 children in the study, 2160 (51.91%) were male and 2001 (48.09%) were female. The median age was 10 years (IQR, 7–13). The serostatus of the children was assessed by the in-house DENV-specific IgG ELISA. Accordingly, the overall seropositivity rate of the cohort was 72.34% (3010/4161), with DENV seropositivity rates gradually rising from 56.93% in children aged 4 to 5 years to 82.44% in those aged 14 to 16 years (Table 1). A significant increase in the age-stratified seropositivity rate was seen (Spearman *r* = 1.0, *P* = .003; Figure 2*A*). The overall seropositivity rate in females was 71.17%, with rates rising from 55.36% in children aged 4 to 5 years to 80% in those aged 14 to 16 years. Similarly, in males, seropositivity rates increased with age, with 58.33% seropositive at 4 to 5 years of age and 84.68% at 14 to 16 years of age, with an overall seropositvity of 73.43% (Supplementary Table 1). However, there was no difference (*P* = .37) in age-stratified seropositivity rates in females vs males (Figure 2*B*) in this cohort.

**Table 1.** Age-Stratified Seropositivity Rates of Children in Gampaha District, Sri Lanka

	Children, No. (%)		
Age, y	Total	Seropositivity	Seronegativity
4–5	613	349 (56.93)	264 (43.07)
6–7	643	418 (65.01)	225 (34.99)
8–9	722	509 (70.50)	213 (29.50)
10–11	687	513 (74.67)	174 (25.33)
12–13	647	521 (80.53)	126 (19.47)
14–16	849	700 (82.45)	149 (17.55)
Total	4161	3010 (72.34)	1151 (27.66)

The presence of dengue virus–specific IgG antibodies was measured in 4161 children aged 4 to 16 years by using an in-house enzyme-linked immunosorbent assay.

### Comparison of the In-house DENV-Specific IgG Assay With the Panbio Assay

As the Panbio assay had been widely used in determining dengue seroprevalence in Sri Lanka and elsewhere [11, 21], we compared the age-stratified seroprevalence rates given by the in-house IgG assay with those of the Panbio assay in a subcohort of children (n = 1374). While 1047 (76.2%) children were shown to be dengue seropositive by the in-house DENV-specific IgG ELISA, the Panbio assay gave a positive result for 941 (68.48%) children, with an equivocal result for 14 children. To assess agreement between the assays (ie, how consistently they classified the same individuals), the Cohen κ statistic was used, and it was 0.33 (95% CI, .27–.38), suggesting fair agreement between the assays in classifying dengue seropositivity. To assess if there was a significant difference in the number of positive results between the assays, the McNemar χ^2^ test showed a significant difference in seropositivity classification between the in-house DENV-specific IgG ELISA and the Panbio assay (χ^2^ = 25.85, *P* < .001), with the in-house assay classifying a greater number of individuals as seropositive. Furthermore, a statistical interaction test via linear regression showed a significant interaction between age and assay type (*P* = .01), indicating that the relationship between age and seroprevalence differs significantly between the Panbio and in-house assays (Figure 2*C*). Comparison of the age-stratified seropositivity rates of the 2 assays showed that with the in-house DENV IgG ELISA, the seropositivity rate was 63.86% among children aged 4 to 5 years, which gradually increased to 82.13% in the 14- to 16-year-old group. However, the Panbio assay showed a lower seropositivity rate of 44.06% at ages 4 to 5 years, which rose progressively to a similar rate of 83.27% at ages 14 to 16 years (Supplementary Table 2).

The sensitivity of the in-house IgG ELISA was 91.8% with a specificity of 75% (Supplementary Methods). In contrast, the sensitivity of the Panbio assay was 83.7% with a specificity of 87.5%. Therefore, the in-house IgG ELISA had higher sensitivity than the Panbio assay in detecting multitypic infections (100% detection), with better correlation with the foci reduction neutralization assay.

Age-stratified seroprevalence was modeled by a bayesian catalytic framework assuming a constant FOI. The estimated FOI was 0.16 (95% credible interval, 0.14–0.17) for the in-house DENV-specific IgG ELISA and 0.13 (95% credible interval, 0.12–0.14) for the commercial Panbio assay, indicating a slightly higher estimated rate of DENV exposure with the in-house assay. The in-house assay, however, showed a less consistent fit under the constant FOI assumption, with the observed seroprevalence in the 8- to 9-year age group notably exceeding model predictions, suggesting that a constant FOI may not fully capture the exposure pattern detected by this assay. This discrepancy likely reflects the higher analytic sensitivity of the in-house ELISA. Despite this limitation, the in-house assay consistently yielded higher seropositivity estimates across all age groups as compared with the Panbio assay, and the model-derived trajectories and 95% credible intervals for both assays supported a meaningfully higher cumulative detection sensitivity for the in-house assay (Figure 2*D*).

### Dengue and Zika Exposure Rates in Children Who Were Dengue Seropositive

Using a Luminex-based bead array, which uses antibodies specific to the EDIII of the 4 serotypes of DENV and the EDIII of ZIKV [20], we proceeded to characterize exposure rates to DENV serotypes and Zika. These assays were carried out in 20% (n = 604) of children who were DENV seropositive, and this subcohort was selected randomly to proportionately represent all age groups and genders of the children included in the study. The number of children tested in each age group for the Luminex assay is shown in Supplementary Table 3. The table shows that the monotypic responses were similar across all age groups, with the lowest being at 33.33% among children who were 6 years old and the highest at ∼55% in those who were 11 and 15 years old.

Of this DENV-seropositive subcohort, 258 (42.7%) had been infected with only 1 DENV in the past (monotypic dengue response), while 209 (34.89%) were infected with many DENV serotypes (multitypic response). Some children who had past monotypic dengue infection were also infected with Zika, and some who had a past multitypic dengue infection had been exposed to Zika (Table 2). Furthermore, 20 (3.33%) children had antibodies to only ZIKV, indicating that the in-house DENV-IgG ELISA could detect cross-reactive flavivirus antibodies. Of the 278 individuals who had a monotypic DENV response and Zika exposure, the main serotype was DENV2 (56.83%), followed by DENV1 (30.57%). Moreover, 37 (6.13%) children who gave a positive response with the in-house DENV IgG ELISA had a negative response with the Luminex assay.

**Table 2.** Exposure to DENV Serotypes and ZIKV in a Subcohort of Children Who Were DENV Seropositive in the Gampaha District

Exposure to DENV or ZIKV	Children Who Tested Positive, No. (%)	Serotype	Response to Each DENV Serotype, No (%)
Monotypic	258 (42.72)	DENV1	78 (30.23)
		DENV2	148 (57.36)
		DENV3	19 (7.36)
		DENV4	13 (5.04)
Monotypic + ZIKV	20 (3.31)	DENV1	7 (35.00)
		DENV2	10 (50.00)
		DENV3	3 (15.00)
		DENV4	0
Multitypic	209 (34.60)		Cannot be determined
Multitypic + ZIKV	60 (9.93)		Cannot be determined
ZIKV	20 (3.31)		Not applicable
Negative	37 (6.13)		Not applicable

The presence of antibodies to DENV serotypes and ZIKV was measured in 604 children who were DENV seropositive by a Luminex assay that uses antigens (EDIII) that can differentiate such antibody responses.

Abbreviations: DENV, dengue virus; ZIKV, Zika virus.

### Differences in Age-Stratified Exposure to DENV Serotypes and ZIKV

**Figure 3.**
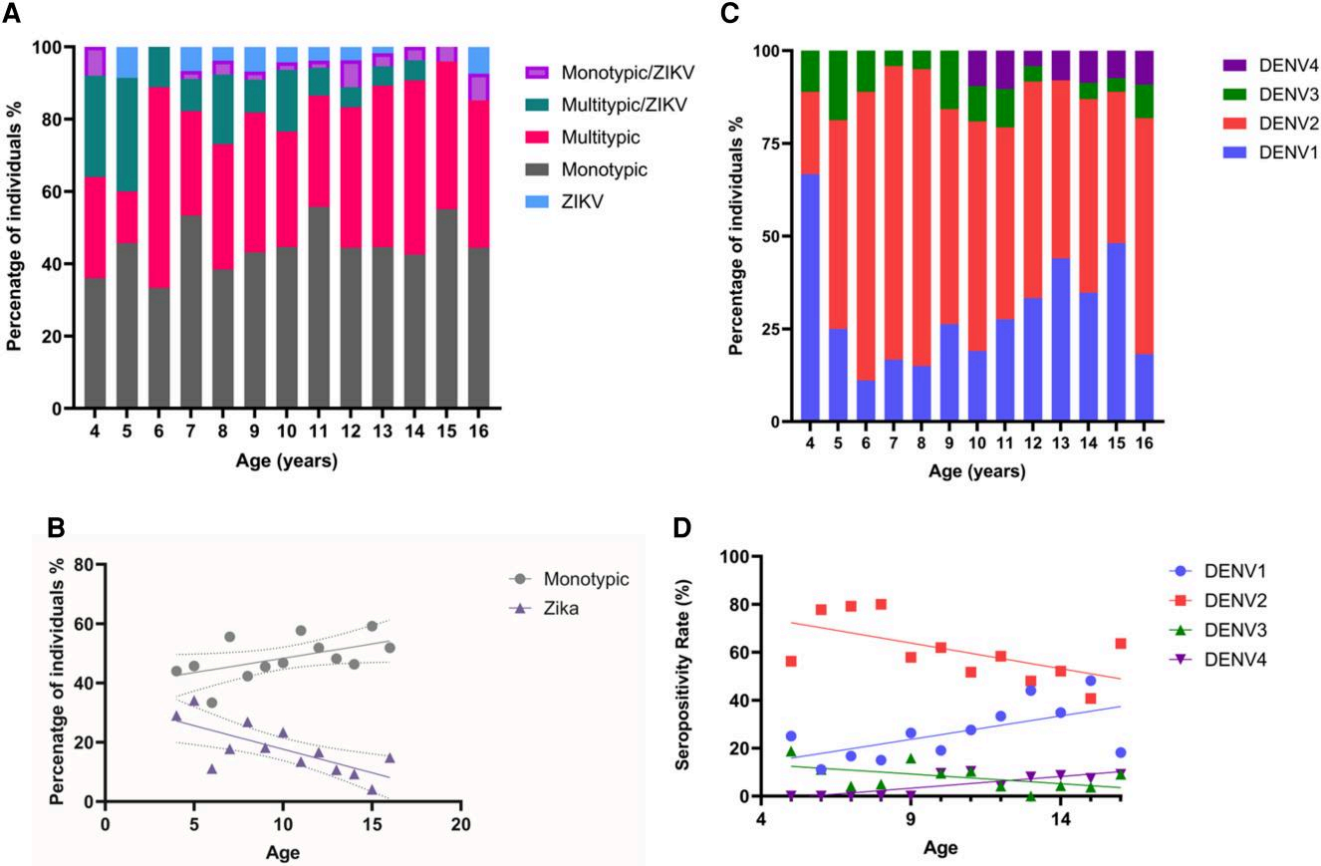
Differences in age-stratified exposure to DENV serotypes and ZIKV. The proportion of children in age groups having a past exposure to only 1 DENV serotype (monotypic response), several DENV serotypes (multitypic infections), ZIKV, or a monotypic or multitypic response with ZIKV was assessed by a multiplexed, microsphere-based serologic assay (Luminex assay). *A*, This assay was carried out on 20% of the seropositive samples (n = 604). *B*, The presence of a monotypic response (gray) or past exposure to ZIKV (blue) was correlated with the age of the children. Dotted lines indicate the 95% confidence intervals. *C*, The multiplexed, microsphere-based serologic assay (Luminex assay) was also used to identify the DENV serotype in those with monotypic responses in age groups. *D*, The exposure to DENV serotypes in children with monotypic responses was correlated with age. The Spearman rank order correlation coefficient was used to evaluate the correlation between age and monotypic and ZIKV past infection rates and between infection rates and DENV serotypes. All tests were 2-sided. DENV, dengue virus; ZIKV, Zika virus.

There was no difference in the proportion of children who had a monotypic vs multitypic response to dengue (Figure 3*A*). However, there was a significant increase (Spearman *r* = 0.62, *P* = .026) in monotypic responses with age, suggesting that many children who were seronegative were continuously exposed to DENV (Figure 2*B*). We also observed an inverse correlation between exposure to ZIKV and age (Spearman *r* = −0.72, *P* = .007; Figure 3*B*). Yet, there were very few children included in the age group of 4 to 5 years as compared with the older groups. There was no difference in the proportion of children who had experienced multitypic infections with age.

Of the children with monotypic responses, DENV2 was the most prominent (Figure 3*C*). DENV4 exposure was not detected in any of the children aged <10 years. While no significant correlation was seen in exposure to DENV2 and DENV3 with age, exposure to DENV1 (Spearman *r* = 0.63, *P* = .03) and DENV4 (Spearman *r* = 0.69, *P* = .01) significantly increased with age (Figure 3*D*).

### Relationship Between Circulating DENV Serotypes and Infections Seen in Children

**Figure 4.**
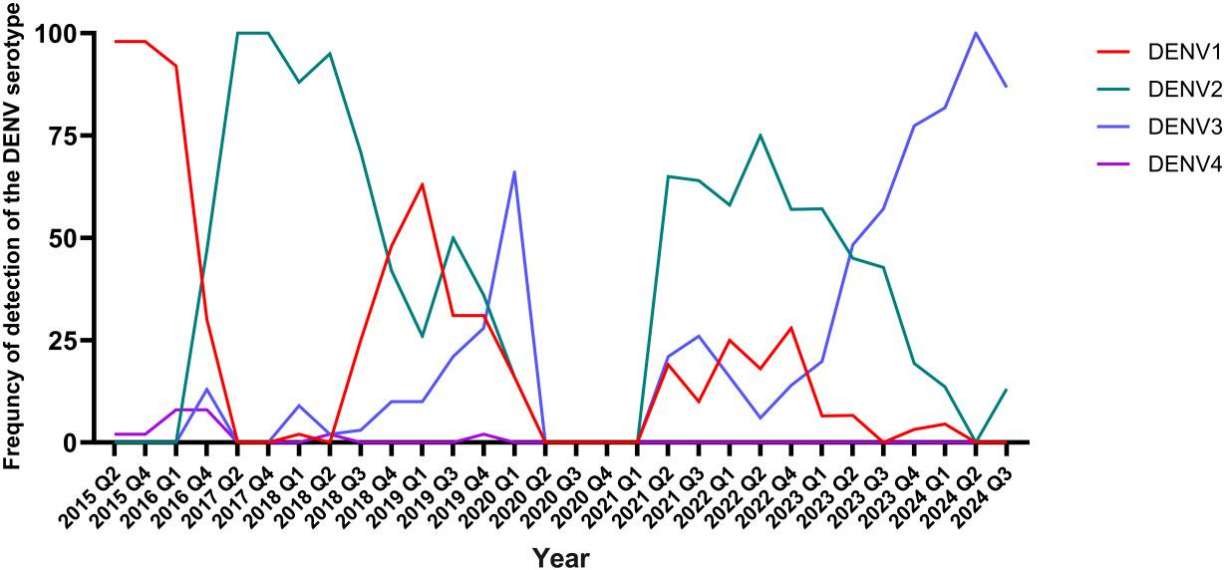
Changes in the circulating DENV serotypes in Colombo, Sri Lanka, from 2015 to January 2024. The proportion of circulating DENV serotypes in the Colombo district is shown over time based on DENV surveillance activities carried out by us over this period [7, 22–24]. The y-axis shows the proportion of circulating DENV as a percentage of all identified serotypes. DENV, dengue virus.

Our laboratory carried out surveillance of DENV serotypes in the Colombo district (adjacent to the Gampaha district) from 2009 until 2024, which has been published [7, 22, 23]. The data from these surveillance activities were used to determine the changes in DENV serotypes throughout this period (Figure 4). Our data show that DENV1 was the predominant serotype from 2009 to 2016 and DENV2 from 2016 to 2019, with DENV3 emerging toward the end of 2019. During 2020 and 2021, the incidence of dengue markedly decreased, and only limited DENV surveillance activities were carried out. Although DENV2 was still the predominant serotype in 2021, it was gradually replaced by DENV3 since 2023 [22]. Because DENV2 was the predominant serotype from 2016 to 2023, it was considered the main serotype causing past infection in children with monotypic dengue responses. DENV1 was seen in 30.57% of children with monotypic responses, as it had been circulating from 2009 and again in recent years. DENV4 had been detected in Colombo until the second quarter of 2018 in 2% to 8% of individuals with dengue infections. It was again detected in the fourth quarter of 2019 in 2.6%. In our cohort, no one aged <10 years with monotypic responses was infected with DENV4 in the past.

## DISCUSSION

In this study, we have determined the age-stratified dengue seroprevalence in a large cohort of children in the Gampaha district, which reports the highest number of dengue infections in Sri Lanka, along with the Colombo district [6]. Our overall seropositivity rate was 72.34%, which was comparable to that in urban populations of Malaysia, Dhakka (Bangladesh), São Paulo (Brazil), and Cambodia but lower than in Thailand, Laos, and the Philippines [10, 25–27]. The estimated FOI was 0.16 for the Gampaha district in this study, which was higher than what we previously reported for Sri Lanka in 2022, where DENV seroprevalence was assessed by the Panbio assay [10, 11]. The Panbio assay has been shown to be less sensitive in detecting previous exposure to DENV, especially in children with a monotypic dengue response, as shown in Cebu, Philippines [28]. In this study, our in-house IgG ELISA was more sensitive than the Panbio assay, and we possibly got higher seropositivity rates than those previously reported. However, the areas chosen for this study were those with the highest dengue incidence in recent years, as assessing the age-stratified seropositivity rates in these areas was carried out as part of a clinical trial to evaluate the effectiveness of a spatial repellent [6]. Therefore, unlike our previous study for assessing age-stratified DENV seroprevalence [11], which included urban, rural, and estate areas, areas reporting the highest incidence were chosen for this study, which might have overestimated the FOI in the whole district.

Females have been shown to have a higher risk of developing severe dengue, and since higher case fatality rates have been reported in females [29–31], we proceeded to determine if there were differences in age-stratified seropositivity rates in females vs males. Age-stratified seropositivity rates were similar in females and males despite the higher severe dengue incidence and mortality among females. The reasons for higher disease severity and mortality in females does not appear to be associated with higher exposure in this group but likely due to “yet to be defined” risk factors or gender-based case management differences. For instance, females develop per vaginal bleeding as a complication, which has shown to associate with an increased risk of dengue hemorrhagic fever and dengue warning signs [32]. Furthermore, gender differences influence disease susceptibility, immune responses to vaccines, and the magnitude and quality of immune responses [33]. Therefore, the increased risk of severe dengue among females could be due to biological factors in addition to social factors, such as delay in seeking health care due to family responsibilities, which should be further investigated.

Although we and others have conducted several studies to determine age-stratified seroprevalence rates in districts in Sri Lanka and over time in Colombo [7, 8, 11], this is the first time that exposure rates to DENV serotypes have been estimated. As such, we were able to define serotypes in primary infections represented by a monotypic response, whereas those with a multitypic response probably represented primarily secondary infections with some primary infections with cross-reactive antibodies. This cohort will be sampled annually to estimate incidence rates to evaluate the protective efficacy of a spatial repellent–based vector control intervention in members who are naive or monotypic because of the difficulty associated with identifying new secondary infections serologically. Among children who were dengue seropositive, 42.7% had experienced only 1 DENV infection in the past, which again indicated higher transmission rates than in northern, western, and eastern regions of India but similar to the trends seen in countries such as Indonesia [34, 35]. Although the seropositivity rate in the children in our study (72.34%) was similar to that seen in southern India in children of the same age (69.6%), most children had a multitypic infection in southern India, indicating higher transmission rates than in our cohort. This highlights the importance of characterizing the proportion of past dengue infections, which are monotypic or multitypic in nature, as it gives more granularity regarding dengue transmission dynamics and the decision regarding vaccine rollout. For instance, TAK-003 has been recommended to be implemented in high-transmission settings, as indicated by a seroprevalence rate >60% by 9 years of age [5]. Therefore, implementation of TAK-003 in the study areas, which had a high incidence of dengue, is likely to reduce the dengue burden, especially given that 42.7% of children who were dengue seropositive had a monotypic response. However, our island-wide seroprevalence studies have shown that the seropositivity rates were <33% in other areas of the same district, and in many areas in Sri Lanka, the seropositivity rates were <20% in 10- to 12-year-olds [11]. Therefore, in such instances, implementation of TAK-003 in certain regions is likely to be beneficial, as opposed to implementation in the whole country.

Due to the highly cross-reactive nature of flavivirus antibodies, the presence of IgG antibodies to DENV [14, 36] could indicate infection with cross-reactive flaviviruses such as ZIKV, West Nile Virus, and Japanese encephalitis virus, which have been shown to circulate in Sri Lanka [16, 37, 38]. Furthermore, seropositivity rates alone do not indicate the exposure rates to DENV, as it would not indicate if an individual has been infected once or several times. In this study, we were able to differentiate between monotypic and multitypic responses and to identify the past infecting serotype in children with monotypic infections, using an assay that detects antibodies to EDIII of DENV and ZIKV, which were shown to be specific with limited cross-reactivity [20]. However, as this Luminex assay detects antibodies only to the EDIII region of DENV and ZIKV, individuals who have lower levels of antibodies to these regions would not be detected, which may be the reason why 6.13% of children who were positive by the in-house IgG ELISA were negative by Luminex. Therefore, this Luminex assay is likely to be less sensitive than the Panbio assay, which detects antibodies to the whole envelope protein, and our in-house IgG ELISA, which detects antibodies to the whole virus. The lower levels of serotype-specific antibodies to EDIII may lead to misclassification of some individuals as having multitypic responses. Yet, as our in-house DENV-IgG ELISA is likely to detect antibodies to the whole DENV, it would have a higher sensitivity but lower specificity due to detection of cross-reactive antibodies, which could be due to previous exposure to ZIKV or Japanese encephalitis virus, which co-circulate in the same region. This is one of the major limitations in flavivirus antibody assays because, as the assay specificity increases by including detection of specific epitopes, the sensitivity is likely to decline.

In this study, we found that DENV2 (56.83%) was the main serotype that had infected most children, followed by DENV1 (30.57%). These findings are consistent with our surveillance data carried out over the years in the Colombo district (adjacent district), which showed that DENV2 was indeed the predominant circulating serotype from 2016 until the end of 2022 [23] (Figure 4). There was a trend toward a reduction of exposure to DENV2 with age, which could be due to individuals being infected with other DENV serotypes resulting in multitypic infections. DENV4 was not identified in our surveillance after 2018, which had been detected in very low frequency in the fourth quarter of 2019. Consistent with our surveillance data, we did not identify children aged <10 years who had been infected with DENV4. DENV3 emerged in 2023, after it had not been detected since 2008, although it was detected for a brief period during the fourth quarter of 2019 [22]. We saw an equal proportion of children exposed to DENV3 in all ages. Therefore, in the absence of past surveillance data, determining exposure rates to DENV serotypes in age groups by these multiplexed, microsphere-based serological assays (Luminex assays) would enable us to characterize the exposure history and get an idea regarding the transmission of DENVs in a particular geographic location in relation to time.

Although we and others had studied the seroprevalence of dengue over many years and in many geographic locations, the seropositivity rates for ZIKV had not been characterized. We found that 16.5% of children also had evidence of past infection with ZIKV, with 3.33% showing evidence of infection with only ZIKV, not DENV, despite the in-house DENV-specific IgG giving a positive result. As virologic surveillance for ZIKV has not been previously carried out in Sri Lanka, there are no data regarding when ZIKV was first introduced to Sri Lanka or how long the virus has been circulating here. However, the first time that ZIKV was identified in Sri Lanka was in patients presenting with dengue-like illness from 2017 to 2018 to a tertiary care hospital [16], which does not obviously indicate that ZIKV was not circulating prior to 2017. In this study, ZIKV was detected in 1% (6/595) of samples by real-time polymerase chain reaction [16]. Interestingly, we saw that infection rates with ZIKV significantly declined with age. Although the reasons for this are not clear, it is possible that prior multitypic infection with DENV reduces the risk of infection with ZIKV. Alternatively, it would be due to the sensitivity of the assays used in detecting ZIKV-specific IgG to EDIII in the context of multitypic responses to DENV. In fact, it has been shown that preexisting higher antibody titers to DENV were associated with a lower risk of symptomatic Zika [39]. As ZIKV infection was seen only in younger children, with lower infection rates of ZIKV in older children, it is likely that ZIKV was recently introduced to Sri Lanka, as older children who were dengue seropositive, possibly with multitypic infections, were protected from ZIKV. Therefore, as the rate of DENV infection increases with age, this could lead to the risk of infection with ZIKV, which would explain our observations. Robust longitudinal cohort investigations incorporating comprehensive virologic and clinical characterization of dengue and Zika infections are essential to determine the extent to which each virus modulates the transmission potential, host susceptibility, and disease manifestations of the other.

In conclusion, we found that 72.3% of children were seropositive for dengue in the Gampaha district in Sri Lanka, which is one of the districts that report the highest number of cases. The dengue seropositivity rate significantly increased with age, although 42.7% of children who were seropositive had been infected with only 1 DENV in the past (monotypic infection). The predominant infecting serotype was DENV2, followed by DENV1, which is consistent with data for the DENV serotype circulating in the last 10 years. Interestingly, infection rates with ZIKV significantly declined with age, suggesting that prior immunity to DENV may reduce the risk of ZIKV infection. These data show that in addition to age-stratified seropositivity data, understanding the proportion of monotypic and multitypic infections and exposure to other flaviviruses is likely to inform better decision making in implementing vaccines and novel vector control strategies.

## Supplementary Material

ofag224_Supplementary_Data

## Supplementary Data

Supplementary materials are available at *Open Forum Infectious Diseases* online. Consisting of data provided by the authors to benefit the reader, the posted materials are not copyedited and are the sole responsibility of the authors, so questions or comments should be addressed to the corresponding author.

## References

[1] The Lancet . Dengue: the threat to health now and in the future. Lancet 2024; 404:311. 39067890 10.1016/S0140-6736(24)01542-3

[2] World Health Organization . Dengue: WHO health emergency appeal 2024. World Health Organization, 2024.

[3] World Health Organization . Pathogens prioritization: a scientific framework for epidemic and pandemic research preparedness. World Health Organization, 2024.

[4] Paz-Bailey G, Adams LE, Deen J, Anderson KB, Katzelnick LC. Dengue. Lancet 2024; 403:667–82. 38280388 10.1016/S0140-6736(23)02576-X PMC12372472

[5] World Health Organization . WHO position paper on dengue vaccines—May 2024. Wkly Epidemiol Rec 2024; 18:203–24.

[6] Tissera H, Dheerasinghe DSAF, Malavige N, et al A cluster-randomized, placebo-controlled trial to evaluate the efficacy of a spatial repellent (mosquito shield) against Aedes-borne virus infection among children >/= 4–16 years of age in the Gampaha District, Sri Lanka: study protocol (the AEGIS program). Trials 2023; 24:9. 36600308 10.1186/s13063-022-06998-z PMC9811041

[7] Malavige GN, Jeewandara C, Ghouse A, Somathilake G, Tissera H. Changing epidemiology of dengue in Sri Lanka—challenges for the future. PLoS Negl Trop Dis 2021; 15:e0009624. 34411101 10.1371/journal.pntd.0009624 PMC8375976

[8] Jeewandara C, Gomes L, Paranavitane SA, et al Change in dengue and Japanese encephalitis seroprevalence rates in Sri Lanka. PLoS One 2015; 10:e0144799. 26696417 10.1371/journal.pone.0144799 PMC4687926

[9] Malavige GN, Fernando S, Aaskov J, et al Seroprevalence of anti-dengue virus antibodies in children in the Colombo district. Dengue Bull 2006; 30:68–71.

[10] Vicco A, McCormack C, Pedrique B, Ribeiro I, Malavige GN, Dorigatti I. A scoping literature review of global dengue age-stratified seroprevalence data: estimating dengue force of infection in endemic countries. EBioMedicine 2024; 104:105134. 38718682 10.1016/j.ebiom.2024.105134 PMC11096825

[11] Jeewandara C, Karunananda MV, Fernando S, et al The burden of dengue in children and risk factors of transmission in nine districts in Sri Lanka. J Med Virol 2024; 96:e29394.

[12] Pushpakumara PD, Jeewandara C, Gomes L, et al Development and validation of an assay for detection of Japanese encephalitis virus specific antibody responses. PLoS One 2020; 15:e0238609. 33112881 10.1371/journal.pone.0238609 PMC7592747

[13] Salgado BB, Maues FCJ, Jordao M, et al Antibody cross-reactivity and evidence of susceptibility to emerging flaviviruses in the dengue-endemic Brazilian Amazon. Int J Infect Dis 2023; 129:142–51. 36736575 10.1016/j.ijid.2023.01.033

[14] Katzelnick LC, Narvaez C, Arguello S, et al Zika virus infection enhances future risk of severe dengue disease. Science 2020; 369:1123–8. 32855339 10.1126/science.abb6143 PMC8274975

[15] Katzelnick LC, Zambrana JV, Elizondo D, et al Dengue and Zika virus infections in children elicit cross-reactive protective and enhancing antibodies that persist long term. Sci Transl Med 2021; 13:eabg9478. 34613812 10.1126/scitranslmed.abg9478 PMC8693842

[16] Ngwe Tun MM, Raini SK, Fernando L, et al Epidemiological evidence of acute transmission of Zika virus infection in dengue suspected patients in Sri-Lanka. J Infect Public Health 2023; 16:1435–42. 37517370 10.1016/j.jiph.2023.07.014

[17] Waafira A, Subbaram K, Faiz R, Un Naher Z, Manandhar PL, Ali S. Zika virus outbreak are on the rise in India: clinical features, complications and prevention. New Microbes New Infect 2024; 62:101493. 39429732 10.1016/j.nmni.2024.101493 PMC11490798

[18] Hasan A, Hossain MM, Zamil MF, et al Concurrent transmission of Zika virus during the 2023 dengue outbreak in Dhaka, Bangladesh. PLoS Negl Trop Dis 2025; 19:e0012866. 39883734 10.1371/journal.pntd.0012866 PMC11813092

[19] Ramu ST, Dissanayake M, Jeewandara C, et al Antibody and memory B cell responses to the dengue virus NS1 antigen in individuals with varying severity of past infection. Immunology 2023; 170:47–59. 37075785 10.1111/imm.13651 PMC11495261

[20] Hein LD, Castillo IN, Medina FA, et al Multiplex sample-sparing assay for detecting type-specific antibodies to Zika and dengue viruses: an assay development and validation study. Lancet Microbe 2025; 6:100951. 39730005 10.1016/j.lanmic.2024.07.014 PMC12244352

[21] Mishra AC, Arankalle VA, Gadhave SA, et al Stratified sero-prevalence revealed overall high disease burden of dengue but suboptimal immunity in younger age groups in Pune, India. PLoS Negl Trop Dis 2018; 12:e0006657. 30080850 10.1371/journal.pntd.0006657 PMC6095695

[22] Ariyaratne D, Senadheera B, Kuruppu H, et al Simultaneous co-circulation of two genotypes of dengue virus serotype 3 causing a large outbreak in Sri Lanka in year 2023. J Infect Dis 2025; 231:1041–48. 39387651 10.1093/infdis/jiae474 PMC11998561

[23] Ariyaratne D, Gomes L, Jayadas TTP, et al Epidemiological and virological factors determining dengue transmission in Sri Lanka during the COVID-19 pandemic. PLOS Glob Public Health 2022; 2:e0000399. 36962516 10.1371/journal.pgph.0000399 PMC10021909

[24] Ariyaratne D, Jayadas TTP, Jeewandara C, et al Molecular epidemiology and evolutionary trends of dengue virus serotype-2 strains in Sri Lanka. BMC Microbiol 2026; 26:91. 10.1186/s12866-025-04584-2. PMC12882535 41449326

[25] Ng RJ, Chong ZL, Abdul Mutalip MH, Ng C-W. Dengue seroprevalence and factors associated with dengue seropositivity in Petaling district, Malaysia. Int J Environ Res Public Health 2022; 19:7170. 35742419 10.3390/ijerph19127170 PMC9223214

[26] Dhar-Chowdhury P, Paul KK, Haque CE, et al Dengue seroprevalence, seroconversion and risk factors in Dhaka, Bangladesh. PLoS Negl Trop Dis 2017; 11:e0005475. 28333935 10.1371/journal.pntd.0005475 PMC5380355

[27] Fox-Lewis A, Hopkins J, Sar P, et al Seroprevalence of dengue virus and rickettsial infections in Cambodian children. Am J Trop Med Hyg 2019; 100:635–8. 30675849 10.4269/ajtmh.18-0865 PMC6402902

[28] Lopez AL, Adams C, Ylade M, et al Determining dengue virus serostatus by indirect IgG ELISA compared with focus reduction neutralisation test in children in Cebu, Philippines: a prospective population-based study. Lancet Glob Health 2021; 9:e44–51. 33212030 10.1016/S2214-109X(20)30392-2 PMC9358663

[29] Anders KL, Nguyet NM, Van Vinh Chau N, et al Epidemiological factors associated with dengue shock syndrome and mortality in hospitalized dengue patients in Ho Chi Minh City, Vietnam. Am J Trop Med Hyg 2011; 84:127–34. 21212214 10.4269/ajtmh.2011.10-0476 PMC3005500

[30] Sangkaew S, Ming D, Boonyasiri A, et al Risk predictors of progression to severe disease during the febrile phase of dengue: a systematic review and meta-analysis. Lancet Infect Dis 2021; 21:1014–26. 33640077 10.1016/S1473-3099(20)30601-0 PMC8240557

[31] Haider N, Asaduzzaman M, Hasan MN, et al Bangladesh's 2023 dengue outbreak—age/gender-related disparity in morbidity and mortality and geographic variability of epidemic burdens. Int J Infect Dis 2023; 136:1–4. 37660728 10.1016/j.ijid.2023.08.026

[32] Wijewickrama A, Kuruppu H, Idampitiya D, et al Per vaginal bleeding—an important but ignored feature of dengue. medRxiv [Preprint]. **2024**. Available from: 10.1101/2024.12.23.24319534

[33] Sharma S, Gibbons A, Saphire EO. Sex differences in tissue-specific immunity and immunology. Science 2025; 389:599–603. 40773572 10.1126/science.adx4381 PMC12777860

[34] Murhekar MV, Kamaraj P, Kumar MS, et al Burden of dengue infection in India, 2017: a cross-sectional population based serosurvey. Lancet Glob Health 2019; 7:e1065–73. 31201130 10.1016/S2214-109X(19)30250-5

[35] Prayitno A, Taurel A-F, Nealon J, et al Dengue seroprevalence and force of primary infection in a representative population of urban dwelling Indonesian children. PLoS Negl Trop Dis 2017; 11:e0005621. 28617803 10.1371/journal.pntd.0005621 PMC5472274

[36] Elong Ngono A, Shresta S. Cross-reactive T cell immunity to dengue and Zika viruses: new insights into vaccine development. Front Immunol 2019; 10:1316. 31244855 10.3389/fimmu.2019.01316 PMC6579874

[37] Lohitharajah J, Malavige GN, Chua AJS, Ng ML, Arambepola C, Chang T. Emergence of human West Nile Virus infection in Sri Lanka. BMC Infect Dis 2015; 15:305. 26227390 10.1186/s12879-015-1040-7 PMC4521480

[38] Lohitharajah J, Malavige N, Arambepola C, et al Viral aetiologies of acute encephalitis in a hospital-based South Asian population. BMC Infect Dis 2017; 17:303. 28438128 10.1186/s12879-017-2403-z PMC5404678

[39] Rodriguez-Barraquer I, Costa F, Nascimento EJM, et al Impact of preexisting dengue immunity on Zika virus emergence in a dengue endemic region. Science 2019; 363:607–10. 30733412 10.1126/science.aav6618 PMC8221194

