# Supplementary material for: Dissecting Age-Stratified Immunity to Different Dengue Virus Serotypes and Zika Viruses Among Children in a Highly Endemic Region in Sri Lanka
Source: Open Forum Infect Dis. 2026 Apr 18;13(5):ofag224. doi: 10.1093/ofid/ofag224 (PMC13143014; doi:10.1093/ofid/ofag224)
Supplement: ofag224_Supplementary_Data [file ofag224_supplementary_data.zip › Supplementary results clean.docx]

**Supplementary results**

| **Age Group** | **Total Number in Each Age Group** | **Males** | | | **Females** | | |
| --- | --- | --- | --- | --- | --- | --- | --- |
|  |  | **Number in Each Age Group (Total Number in each age group)** | **Seropositivity Rate**  **N (%)** | **Sero-negativity Rate**  **N (%)** | **Number in Each Age Group (Total Number in each age group)** | **Seropositivity Rate**  **N (%)** | **Sero-negativity Rate**  **N (%)** |
| 4-5 | 613 | 324 (613) | 189 (58.33%) | 135(41.67%) | 289 (613) | 160 (55.36%) | 129 (44.63%) |
| 6-7 | 643 | 341 (643) | 217 (63.36%) | 124 (36.36%) | 302 (643) | 201 (66.56%) | 101 (33.44%) |
| 8-9 | 722 | 369 (722) | 262 (71.10%) | 107 (29.00%) | 353 (722) | 247 (70.00%) | 106 (30.55%) |
| 10-11 | 687 | 340 (687) | 261 (76.77%) | 79 (23.23%) | 347 (687) | 252 (72.62%) | 95 (27.38%) |
| 12-13 | 647 | 342 (647) | 281 (82.16%) | 61 (17.84%) | 305 (647) | 240 (78.69%) | 65 (21.31%) |
| 14-16 | 849 | 444 (849) | 376 (84.68%) | 68 (18.10%) | 405 (849) | 324 (80.00%) | 81 (20.00%) |
| **Total** | **4161** | **2160 (4161)** | **1586 (73.43%)** | **574 (26.57%)** | **2001 (4161)** | **1424 (71.17%)** | **577 (28.84%)** |

**Supplementary table 1: The seropositivity rates for dengue among males and females of different age groups**

| **Total Seropositivity Rates** | **Total Number in Each Age Group** | **In house DENV IgG ELISA** | | **Panbio Indirect Dengue IgG ELISA** | |  |
| --- | --- | --- | --- | --- | --- | --- |
| **Age Group (years)** |  | **Seropositivity Rate n (%)** | **Seronegativity Rate n (%)** | **Seropositivity Rate n (%)** | **Equivocal Rate n (%)** | **Seronegativity Rate n (%)** |
| 4-5 | 202 | 129 (63.86%) | 73 (36.14%) | 89 (44.06%) | 2 (0.99%) | 111 (54.95%) |
| 6-7 | 221 | 162 (73.30%) | 59 (26.70%) | 137 (61.99%) | 1 (0.45%) | 83 (37.56%) |
| 8-9 | 243 | 188 (77.37%) | 55 (22.63%) | 165 (67.90%) | 1 (0.41%) | 77 (31.69%) |
| 10-11 | 235 | 184 (78.30%) | 51 (21.70%) | 164 (69.79%) | 2 (0.85%) | 69 (29.36%) |
| 12-13 | 210 | 168 (80.00%) | 42 (20.00%) | 167 (79.52%) | 5 (2.38%) | 38 (18.10%) |
| 14-16 | 263 | 216 (82.13%) | 47 (17.87%) | 219 (83.27%) | 3 (1.14%) | 41 (15.59%) |
| **Total** | **1374** | **1047 (76.20%)** | **327 (23.80%)** | **941 (68.49%)** | **14 (1.01%)** | **419 (30.49%)** |

**Supplementary table 2: Age stratified Seropositivity and Seronegativity rates in children in the two assays. Comparison of the seropositivity rates and seronegativity rates of between the In house DENV IgG ELISA and the Panbio ELISA with age.**

| Age | Antigen | Number | Total | Percentages |
| --- | --- | --- | --- | --- |
| 4 | Monotypic | 9 |  | 36.00 |
|  | Multitypic | 7 |  | 28.00 |
|  | Multitypic/ZIKV | 7 |  | 28.00 |
|  | Monotypic/ZIKV | 2 |  | 8.00 |
|  | ZIKV | 0 |  | 0.00 |
|  | Negative | 6 | 31 | 24.00 |
| 5 | Monotypic | 16 |  | 45.71 |
|  | Multitypic | 5 |  | 14.29 |
|  | Multitypic/ZIKV | 11 |  | 31.43 |
|  | Monotypic/ZIKV | 0 |  | 0.00 |
|  | ZIKV | 3 |  | 8.57 |
|  | Negative | 6 | 41 | 17.14 |
| 6 | Monotypic | 9 |  | 33.33 |
|  | Multitypic | 15 |  | 55.56 |
|  | Multitypic/ZIKV | 3 |  | 11.11 |
|  | Monotypic/ZIKV | 0 |  | 0.00 |
|  | ZIKV | 0 |  | 0.00 |
|  | Negative | 5 | 32 |  |
| 7 | Monotypic | 24 |  | 53.33 |
|  | Multitypic | 13 |  | 28.89 |
|  | Multitypic/ZIKV | 4 |  | 8.89 |
|  | Monotypic/ZIKV | 1 |  | 2.22 |
|  | ZIKV | 3 |  | 6.67 |
|  | Negative | 5 | 50 | 11.11 |
| 8 | Monotypic | 20 |  | 38.46 |
|  | Multitypic | 18 |  | 34.62 |
|  | Multitypic/ZIKV | 10 |  | 19.23 |
|  | Monotypic/ZIKV | 2 |  | 3.85 |
|  | ZIKV | 2 |  | 3.85 |
|  | Negative | 2 | 54 | 3.85 |
| 9 | Monotypic | 19 |  | 43.18 |
|  | Multitypic | 17 |  | 38.64 |
|  | Multitypic/ZIKV | 4 |  | 9.09 |
|  | Monotypic/ZIKV | 1 |  | 2.27 |
|  | ZIKV | 3 |  | 6.82 |
|  | Negative |  | 44 | 0.00 |
| 10 | Monotypic | 21 |  | 44.68 |
|  | Multitypic | 15 |  | 31.91 |
|  | Multitypic/ZIKV | 8 |  | 17.02 |
|  | Monotypic/ZIKV | 1 |  | 2.13 |
|  | ZIKV | 2 |  | 4.26 |
|  | Negative | 4 | 51 | 8.51 |
| 11 | Monotypic | 29 |  | 55.77 |
|  | Multitypic | 16 |  | 30.77 |
|  | Multitypic/ZIKV | 4 |  | 7.69 |
|  | Monotypic/ZIKV | 1 |  | 1.92 |
|  | ZIKV | 2 |  | 3.85 |
|  | Negative | 4 | 56 | 7.69 |
| 12 | Monotypic | 24 |  | 44.44 |
|  | Multitypic | 21 |  | 38.89 |
|  | Multitypic/ZIKV | 3 |  | 5.56 |
|  | Monotypic/ZIKV | 4 |  | 7.41 |
|  | ZIKV | 2 |  | 3.70 |
|  | Negative | 1 | 55 | 1.85 |
| 13 | Monotypic | 25 |  | 44.64 |
|  | Multitypic | 25 |  | 44.64 |
|  | Multitypic/ZIKV | 3 |  | 5.36 |
|  | Monotypic/ZIKV | 2 |  | 3.57 |
|  | ZIKV | 1 |  | 1.79 |
|  | Negative | 1 | 57 | 1.79 |
| 14 | Monotypic | 23 |  | 42.59 |
|  | Multitypic | 26 |  | 48.15 |
|  | Multitypic/ZIKV | 3 |  | 5.56 |
|  | Monotypic/ZIKV | 2 |  | 3.70 |
|  | ZIKV | 0 |  | 0.00 |
|  | Negative | 1 | 55 | 1.85 |
| 15 | Monotypic | 27 |  | 55.10 |
|  | Multitypic | 20 |  | 40.82 |
|  | Multitypic/ZIKV | 0 |  | 0.00 |
|  | Monotypic/ZIKV | 2 |  | 4.08 |
|  | ZIKV | 0 |  | 0.00 |
|  | Negative | 2 | 51 | 4.08 |
| 16 | Monotypic | 12 |  | 40.74 |
|  | Multitypic | 11 |  | 40.74 |
|  | Multitypic/ZIKV | 0 |  | 0.00 |
|  | Monotypic/ZIKV | 2 |  | 7.41 |
|  | ZIKV | 2 |  | 7.41 |
|  | Negative | 0 | 27 | 0.00 |

**Supplementary table 3: Exposure to different DENV serotypes and Zika in a sub-cohort of children who were DENV seropositive in the Gampaha district. The presence of antibodies to different DENV serotypes and ZIKV was measured in 604 children who were DENV seropositive by using a Luminex assay which uses antigens (EDIII), which can differentiate antibody responses to different DENV serotypes and ZIKV.**
